# Subcutaneous vedolizumab interval extension in inflammatory bowel disease patients: a case series

**DOI:** 10.1177/17562848241228080

**Published:** 2024-02-24

**Authors:** Suzanne I. Anjie, Krisztina B. Gecse, Cyriel Y. Ponsioen, Mark Löwenberg, Geert R. D’Haens

**Affiliations:** Department of Gastroenterology and Hepatology, Amsterdam University Medical Centres, Amsterdam, The Netherlands; Department of Gastroenterology and Hepatology, Amsterdam University Medical Centres, Amsterdam, The Netherlands; Department of Gastroenterology and Hepatology, Amsterdam University Medical Centres, Amsterdam, The Netherlands; Department of Gastroenterology and Hepatology, Amsterdam University Medical Centres, Amsterdam, The Netherlands; Department of Gastroenterology and Hepatology, Amsterdam University Medical Centres, Meibergdreef 9, Amsterdam 1105 AZ, The Netherlands

**Keywords:** case series, interval extension, personalized medicine, vedolizumab

## Abstract

Subcutaneous vedolizumab has demonstrated efficacy as a maintenance therapy in inflammatory bowel disease (IBD). However, data on the extension of subcutaneous vedolizumab injection intervals are lacking. Here, we present the first real-world data on subcutaneous vedolizumab interval extension in IBD patients. Nine patients (eight Crohn’s disease patients and one ulcerative colitis patient) were included in the study. At interval extension (at baseline), all patients were in clinical and biochemical remission and requested an extension of their 2-weekly injection intervals due to side effects potentially related to subcutaneous vedolizumab. Patients increased their intervals to 3, 4, or 5 weeks. During a median follow-up of 10.0 months (IQR 6.5–19.5), no flare-ups were observed. After 6 months, median biochemical parameters remained stable compared to baseline levels (fecal calprotectin 24.0 µg/g [IQR 10.0–43.0] versus 28.0 µg/g [IQR 15.0–54.0], *p* = 0.553; C-reactive protein 3.4 mg/L [IQR 1.4–4.2] versus 3.1 mg/L [IQR 0.7–4.9], *p* = 0.172), while vedolizumab serum concentrations significantly decreased (22.0 µg/mL [IQR 20.0–33.0] *versus* 40.0 µg/mL [IQR 28.3–45.0], *p* = 0.018). After interval extension, almost all suspected vedolizumab-induced side effects disappeared within 6 months. Lengthening subcutaneous vedolizumab intervals in IBD patients in clinical and biochemical remission appears to be both effective and safe, potentially leading to substantial reductions in healthcare expenses.

## Plain language summary

Extending subcutaneous vedolizumab injection intervals in patients with inflammatory bowel disease: a case series

We observed nine patients with inflammatory bowel disease who extended the time between injections of subcutaneous vedolizumab. All patients initially received subcutaneous vedolizumab every two weeks and were in clinical and biochemical remission. However, they wanted to extend the injection interval due to possible side effects. They gradually increased their injection intervals to 3, 4, or 5 weeks. Over a median follow-up of 10 months, none of the patients experienced a flare-up. After six months, clinical and biochemical parameters remained stable, while vedolizumab serum concentrations decreased. Side effects that may have been caused by vedolizumab mostly resolved within six months of extending the injection intervals. Lengthening the time between subcutaneous vedolizumab injections for patients in remission appears to be effective, safe, and may also reduce healthcare costs.

## Introduction

Subcutaneous (SC) vedolizumab (VDZ) is an effective maintenance therapy in inflammatory bowel disease (IBD).^1,2^ VDZ is a gut-selective humanized IgG1 monoclonal antibody that specifically binds to α4β7 integrins. After intravenous (IV) induction treatment with at least two infusions of 300 mg VDZ, the recommended dose of SC VDZ maintenance treatment is 108 mg once every 2 weeks. Up until now, no data are available on the extension of SC VDZ injection intervals. We here report the first real-world data of SC VDZ interval extension in IBD.

## Case series

This retrospective study was conducted at a tertiary referral center. Patients were identified using pharmacy records; each electronic medical record was reviewed manually. The reporting of this study complies with the CARE statement.^3^

Nine patients were included in the study, comprising eight Crohn’s disease patients and one ulcerative colitis patient. The median age at interval lengthening (baseline) was 55.0 years (interquartile range [IQR] 44.5–62.0), with a median disease duration of 16.0 years (IQR 9.5–23.5). Eight out of nine patients (89%) received previous advanced therapy, including infliximab, adalimumab, and ustekinumab. Montreal classification of Crohn’s disease patients was distributed as follows: 6/8 A2 (age at onset 17–40 years), 2/8 A3 (age at onset >40 years), 4/8 L1 (disease location restricted to ileum), 2/8 L2 (colonic disease), 2/8 L3 (ileocolonic disease), 4/8 B1 (inflammatory phenotype), 2/8 B2 (stricturing phenotype), and 2/8 B3 (penetrating phenotype). Five Crohn’s disease patients underwent IBD-related surgery prior to SC VDZ de-escalation, 4/8 underwent an ileocecal resection, one of these patients also underwent a subsequent small bowel resection, and one patient underwent a proctocolectomy with permanent ileostomy. The majority (6/9) of patients switched to SC VDZ immediately after IV VDZ induction treatment, of the three remaining patients mean time from switching from IV to SC VDZ was 77 months (standard deviation 14). At baseline, all patients were in clinical (i.e. Harvey–Bradshaw index <5 for Crohn’s disease patients and simple clinical colitis activity index <4 for ulcerative colitis patients) and biochemical (i.e. median fecal calprotectin levels were 28.0 µg/g [IQR 15.0–54.0], and all values were below 150 µg/g) remission for at least 6 months (Table 1). One patient used budesonide for the past 2 years and none of the patients received treatment with concomitant aminosalicylates, immunomodulators, biologics, or small molecules at baseline.

**Table 1.** Baseline characteristics of individual patients including disease activity parameters, vedolizumab serum concentrations, adverse events which were reasons to de-escalate subcutaneous vedolizumab, and corresponding scores 6 months after de-escalation.

Patient	A	B	C	D	E	F	G	H	I
Baseline characteristics									
Clinical score	SCCAI = 0	HBI = 1	HBI = 0	HBI = 2	HBI = 0	HBI = 2	HBI = 1	HBI = 0	HBI = 1
C-reactive protein (mg/L)	2.9	3.3	0.3	4.3	5.2	N/A	0.6	1.0	5.2
Fecal calprotectin (µg/g)	52	11	20	44	84	56	28	18	12
VDZ serum concentration (µg/mL)	45	29	33	44	28	45	24	49	40
Adverse event(s)	Headache and arthralgia	Fatigue	Injection site reaction	Frequent respiratory and skin infections	Fatigue	Constipation	Headache	Arthralgia	Fatigue
Six months after subcutaneous vedolizumab de-escalation									
SC VDZ dosing interval	Q4W	Q3W	Q3W	Q3W	Q3W	Q3W	Q3W	Q3W	Q3W
Clinical score	SCCAI = 0	HBI = 1	HBI = 0	HBI = 2	HBI = 1	HBI = 1	HBI = 0	HBI = 0	HBI = 0
C-reactive protein (mg/L)	1.4	4.1	N/A	3.0	3.7	N/A	N/A	1.4	4.5
Fecal calprotectin (µg/g)	43	9	N/A	17	111	43	N/A	10	24
VDZ serum concentration (µg/mL)	22	21	16	N/A	20	40	N/A	33	22

HBI, Harvey–Bradshaw index; N/A, not available; QXW, every X weeks; SCCAI, simple clinical colitis activity index; SC, subcutaneous; VDZ, vedolizumab.

All patients requested their treating physician to extend the injection interval because of side effects, possibly related to VDZ, which included fatigue (3/9), headache (2/9), arthralgia (2/9), recurrent respiratory and skin infections (1/9), constipation (1/9), and injection site reactions (1/9). SC VDZ dosing intervals were prolonged in a step-wise manner to every 3 (8/9) or every 4 weeks (1/9), and in two patients eventually to every 5 weeks.

All patients were routinely assessed at our outpatient clinic with regularly scheduled laboratory evaluations. The median follow-up time was 10.0 months (IQR 6.5–19.5). During follow-up, no increase in IBD-related complaints suggesting a flare was observed. Six months after SC VDZ interval extension, median fecal calprotectin levels remained stable compared to baseline [24.0 µg/g (IQR 10.0–43.0), *p* = 0.553] and remained below the 150 µg/g cutoff level. Six months after interval extension, C-reactive protein serum levels did not differ from baseline measurements [3.4 mg/L (IQR 1.4–4.2) *versus* 3.1 mg/L (IQR 0.7–4.9), *p* = 0.172]. No patient switched back to a 2-week SC injection interval. During follow-up, two surveillance colonoscopies were performed in two Crohn’s disease patients, confirming mucosal healing (i.e. total simple endoscopic score for Crohn’s disease score of zero). All the symptoms, that initially led to interval extension, except for fatigue, disappeared within 6 months after implementing the new dosing scheme.

In total, 25 VDZ serum concentrations were measured during follow-up, two patients had no follow-up VDZ serum concentration. Median VDZ concentrations at baseline were 40.0 µg/mL (IQR 28.3–45.0) and dropped to 22.0 µg/mL (IQR 20.0–33.0), *p* = 0.018, 5–7 months thereafter. Anti-drug antibodies against VDZ were not evaluated but all serum concentrations were above 12 µg/mL, from which the absence of clinically relevant neutralizing anti-drug antibodies against VDZ could be inferred.^4^

## Discussion

Our findings indicate that SC VDZ interval extension to every 3, 4, or even every 5 weeks did not result in clinical and/or biochemical relapse in patients who were in stable remission, despite significantly decreased VDZ serum concentrations.

All patients were de-escalated due to adverse events possibly caused by VDZ. Intriguingly, after interval extension, all suspected VDZ-induced side effects disappeared within 6 months, except for fatigue. These observations suggest that these manifestations may be dose-related, and patients experiencing commonly reported adverse events could potentially benefit from SC VDZ interval extension, leading to improved drug tolerability without losing efficacy.

Given the rise in IBD-related healthcare costs, which is mainly caused by increasing expenses of expensive advanced therapeutic agents that currently constitute 50–75% of the total IBD-associated costs, it is essential to develop cost-effective strategies.^5^ Interval extension of SC VDZ administration may offer a promising approach to reduce the financial burden on hospitals, payers, and society, by potentially decreasing the costs of these biologic treatments.

One retrospective study analyzed the risk of clinical relapse in 34 patients with IBD undergoing interval extension of IV VDZ.^6^ Patients with quiescent IBD extended their dosing interval from 4 to every 8 weeks. During a 2-year follow-up period, a relapse rate of 15% was found. Remission could be recaptured in 80% of these patients when the treatment interval of IV VDZ was decreased in case of a flare. Due to differences in pharmacokinetic aspects, such as bioavailability, and higher and more stable serum concentrations with a different area under the curve, these findings may not be directly extrapolated to subcutaneously treated patients. Our retrospective analysis of de-escalated SC VDZ IBD patients provides evidence that the dosing interval could be safely extended with the SC formulation. Because no disease flares were observed in our cohort, we could not study the recapture phenomenon. Of note, the potential risks and benefits of SC VDZ de-escalation should be balanced on a case-by-case basis, particularly until long-term follow-up data of SC VDZ interval extension and recaptured responses following treatment intensification in case of a disease flare becomes available.

Currently, target serum concentration or cutoff levels for SC VDZ are lacking to guide clinicians when considering injection interval extension without causing a relapse. Therefore, more data are needed to better understand the role of therapeutic drug monitoring when VDZ injection intervals are extended.

This study has some limitations. There were some missing data because of the retrospective study design. However, due to strict follow-up of patients, which included regular laboratory check-ups, missing data were minimized. Moreover, the small sample size limits powered analysis and some of the patients had a relatively short follow-up. On the other hand, an earlier VDZ de-escalation study described a median time to relapse of 14 weeks and therefore our follow-up time might be sufficient.^6^

Although larger prospective studies with longer follow-ups are needed, these first real-world data of SC VDZ interval extension in IBD patients in clinical and biochemical remission point in the direction that this is an effective and safe approach, that could potentially result in significantly decreased healthcare expenses.

## Declarations

**Ethics approval and consent to participate:** Patients stated that information from the electronic medical records may be used for research purposes and/or agreed to participate via verbal or written informed consent.

**Consent for publication:** Patients stated that information from the electronic medical records may be used for publication.

**Availability of data and materials:** The data underlying this article cannot be shared publicly due to the privacy of individuals who participated in the study.
